# Does Optilume drug-coated balloon dilation compromise female sphincter function?

**DOI:** 10.1007/s11255-025-04513-2

**Published:** 2025-04-17

**Authors:** Lukas Andrius Jelisejevas, Gennadi Tulchiner, Patricia Kink, Peter Rehder

**Affiliations:** Department of Urology, Medical University Innsbruck, Anichstrasse 35, 6020 Innsbruck, Austria

**Keywords:** Drug-coated balloon dilation, Paclitaxel, Female urethral stricture, Urethroplasty, Urethral reconstruction, Direct vision internal urethrotomy

## Abstract

**Purpose:**

Female urethral strictures (FUS) are rare, yet when present very bothersome. Repeated dilation often results in recurrence, requiring surgical urethral reconstruction in the long term. We assessed the outcomes of female patients with urethral stricture disease undergoing Optilume drug-coated-balloon (DCB) dilation.

**Methods:**

Between August 2021 and May 2024, 14 female patients with FUS underwent DCB dilation. Two transgender male-to-female patients were excluded. The diagnosis was confirmed by inability to pass a 14Fr catheter, translabial ultrasound or voiding cystourethrogram (VCUG). Exclusion criteria were urinary tract infection, neurological disease and previous urethroplasty. Written consent was obtained prior to intervention and included the option of urethroplasty. After hydrophilic urethral dilation, urethrocystoscopy was performed. DCB (5 cm) dilation was done to 30Fr at 10 bar for 10 min. General or local anaesthesia was used depending on patient’s preference. Transurethral catheter was placed for 2–5 days. Endpoints were urinary continence, anatomical success (≥ 14Fr by cystoscopy/calibration) and freedom from repeat intervention at 6 months.

**Results:**

The mean age was 52.3 years (range: 31–81), the mean number of prior interventions was 4.3 (range: 0–40). The mean follow-up was 12 months (range: 6–41). At the time of analysis, 11 out of 12 subjects (91.7%) were free from recurrence and repeat intervention. All patients remained continent after the intervention, with two reporting complete resolution of preexisting incontinence. Limitations of the study include the small sample size, short follow-up and single centre nature.

**Conclusions:**

Optilume DCB dilation demonstrates short-term efficacy, is well tolerated, safe, and notably preserves sphincter function.

## Introduction

Female urethral stricture (FUS) is a rare condition that significantly affects patients’ quality of life. It is defined as anatomical narrowing causing reduced urethral lumen. Prevalence is not well defined and most likely underestimated. Reports indicate that 4 to 20% of female patients with bladder outlet obstruction (BOO) are diagnosed with urethral stricture disease [1]. Its aetiology is diverse, encompassing iatrogenic injury, trauma, inflammation, and idiopathic causes [2]. Urethral dilation represents the first-line treatment and is associated with high recurrence rates [3]. Repeat dilation yields worse patency rates and intermittent self-dilation (ISD) is often required [4]. Urethroplasty is recommended in women with a second recurrence of FUS who wish definitive treatment or cannot perform ISD [5].

In December 2021, the Optilume drug-coated balloon (DCB) (Laborie, Plymouth, MN, USA) received FDA approval for managing recurrent anterior male urethral strictures [6]. It combines mechanical dilation with localised delivery of antiproliferative agent paclitaxel. It represents an innovative approach to address the challenges of recurrent urethral strictures by targeting the underlying pathophysiology of fibrosis. Whilst the efficacy of DCB has been demonstrated in treatment of anterior urethral strictures in male patients [7], evidence regarding its application in female patients remain limited [8]. This study aims to evaluate the effect of Optilume DCB dilation on sphincter function in female patients with FUS.

## Methods

### Study population

Between August 2021 and May 2024, 14 female patients with FUS underwent Optilume DCB dilation at a single tertiary centre. Two transgender male-to-female (MtF) patients were excluded. General anaesthesia was administered to eight subjects, whilst four underwent the procedure under local anaesthesia using urethral anaesthetic gel. The diagnosis was confirmed, and the stricture length was measured using a combination of methods: inability to pass a 14Fr catheter, translabial ultrasound, voiding cystourethrogram (VCUG) and the passage of a scaled cystoscope sheath. A thorough clinical pelvic examination was performed in each case. Five patients underwent additional videourodynamic testing. All postmenopausal patients had undergone local oestrogen therapy prior to the intervention. Exclusion criteria were urinary tract infection, neurological disease, previous urethroplasty and external meatal stenosis. Written consent was obtained prior to treatment and included the option of contemporary female urethroplasty. The present study was approved by the institutional review board of the Medical University Innsbruck (Study number: 1101/2022).

### Surgical procedure and follow-up

After urethral dilation with hydrophilic dilators, urethrocystoscopy was performed to assess the urethra and exclude other pathologies. The procedure was performed under either local or general anaesthesia, depending on the patient’s preference. The 30Fr Optilume DCB (5 cm) was advanced into the urethra, ensuring that 0.5 cm of the drug-coated segment of the balloon remained exposed externally. Dilation was performed by inflating the balloon to 30Fr, 10 bar for 10 min. A 14Fr Foley catheter was placed for 25 days. We advised the patients to abstain from sexual activity for 2 weeks and use barrier contraception for 3 months. Follow-up was performed at 4 weeks, 3 months and then every 3 months.

### Study endpoint and statistical analysis

The endpoints were urinary continence, anatomical success (≥ 14Fr by cystoscopy/calibration) and freedom from repeat intervention at 6 months. The International Consultation on Incontinence Questionnaire-Urinary Incontinence Short Form (ICIQ-UI SF) was used before the surgery and at 6 months to assess urinary continence. The Patient Global Impression of Improvement (PGI-I) assessment was utilised at 6 months to evaluate patients’ perceptions of improvement following surgery. Statistical analysis was performed with SPSS Version 28.

## Results

The mean age was 52.3 years (range: 31–81), the mean number of prior interventions was 4.3 (range: 0–40). The mean follow-up was 12 months (range: 6–41). Further characteristics are presented in Table 1. At the time of analysis, 11 out of 12 subjects (91.7%) were recurrence-free. At 6 months, all 11 patients rated their improvement as “very much better” using the PGI-I assessment.

**Table 1:** Descriptive characteristics for 12 female patients treated with Optilume DCB

Parameter	Result, [range]
Age (yrs)	52.3 [31–81]
Body mass index (kg/m2)	24.9 [19.7–37.2]
Prior interventions	4.3 [0–40]
Smoker	
Yes	2 (16.7%)
No	10 (83.3%)
Coronary artery disease	
Yes	1 (8.3%)
No	11 (91.7%)
Diabetes	
Yes	0 (0%)
No	12 (100%)
Irradiation	
Yes	0 (0%)
No	12 (100%)
Stricture length (mm)	
≤ 5	4 (33.3%)
5–10	6 (50%)
> 10	2 (16.7%)
Stricture location	
Proximal	3 (25%)
Mid-urethral	6 (50%)
Distal	2 (16.7%)
Panurethral	1 (8.3%)
Aetiology	
Iatrogenic	6 (50%)
Idiopathic	2 (16.7%)
Lichen sclerosus	2 (16.7%)
Trauma	2 (16.7%)
Number of prior interventions	4.3 [0–40]
Follow-up (months)	12 [6–41]

No new cases of incontinence or adverse events were observed. Before surgery, two patients reported experiencing urinary incontinence. One patient experienced significant residual urine volume with overflow incontinence, which resolved following successful bladder emptying the intervention. The second patient had symptoms of overactive bladder with urge incontinence, which we believe was secondary to obstruction and improved after the intervention.

## Discussion

The use of Optilume DCB dilation for the treatment of FUS represents a promising and innovative approach. The procedure is well tolerated, safe, and preserves sphincter function. It can be conveniently performed in an outpatient setting under local anaesthesia. Notably, urethral dilation up to 30Fr did not compromise sphincter integrity, maintaining urinary continence in all 12 patients.

Reports suggest that paclitaxel, after intravenous application, is excreted into breast milk and reaches levels below the minimum quantifiable concentrations at approximately 72 h after application [9]. However, no comparable data exist for our intended use. We advised all patients to abstain from sexual activity for 2 weeks and use barrier contraception for 3 months. None of the patients were breastfeeding nor trying to become pregnant. Whilst the short-term results are encouraging, further studies are planned to evaluate long-term outcomes of urethral patency. The study’s limitations include its small sample size, short follow-up period, and single-centre design.

## Conclusions

Dilation of the female urethra with a DCB to 30Fr for 10 min does not compromise sphincter function. In cases of a tight stricture, we recommend careful predilation to at least 20Fr, to prevent tear-formation through the urethral wall and/or sphincter mechanism.

## Data Availability

No datasets were generated or analysed during the current study.
